# Recent FDA-approved kinase inhibitors for cancer therapy in 2025: A comprehensive review and perspectives

**DOI:** 10.17179/excli2025-8972

**Published:** 2025-11-14

**Authors:** Mateen Abbas, Syed Hassam Ali Sami, Márió Gajdács, Muhammad Junaid Tariq

**Affiliations:** 1 Department of Pharmacy Practice, Faculty of Pharmacy, Capital University of Science and Technology, Islamabad, Pakistan; 2 Department of Oral Biology and Experimental Dental Research, Faculty of Dentistry, University of Szeged, 6720 Szeged, Hungary; 3 Department of Pharmaceutical Chemistry, Faculty of Pharmacy, Capital University of Science and Technology, Islamabad, Pakistan

**Keywords:** cancers, FDA approvals, kinase inhibitors, anticancer therapy, literature review

## Abstract

Malignant disorders continue to represent one of the major burdens of disease globally, especially in the context of premature deaths. Targeted anticancer treatments, including kinase inhibitors (KIs), have become crucial tools to disrupt the specific signaling pathways that are responsible for cancer growth following malignant transformation. Evidence demonstrates that KIs have substantially advanced precision oncology across multiple malignancies, with clinical success most notable in hematologic cancers and specific solid tumors, such as non-small cell lung cancer. Nonetheless, their long-term efficacy is often constrained by the emergence of acquired resistance, intratumoral heterogeneity, and off-target toxicities, underscoring the need for adaptive therapeutic strategies and combination regimens. While next-generation KIs and ongoing trials of KIs have the promise to expand the therapeutic landscape, the uneven distribution of clinical benefits across different cancer types reveals a considerable gap between molecular advances and real-world outcomes, leading to unequitable improvements in survival and quality of life for patients. Research also indicates disparities in access and affordability, raising concerns about their integration into routine care in low- and middle-income countries. The present review paper aims to provide a summary and a critical synthesis of the development, therapeutic potential, and clinical performance of novel of kinase inhibitors in oncology (i.e. zongeritinib, sunvozertinib, vimseltinib, mirdametinib, avutometinib and defactinib), authorized by the US Food and Drug Administration (FDA) in 2025, aiming to highlight both their transformative role and their inherent limitations. Taken together, KIs represent both a milestone and a challenge in oncology: they highlight the success of rational drug design and targeted therapy, yet show the need for continual innovation, improved global accessibility, and integration into multimodal strategies and standards of care to achieve durable survival benefits.

See also the graphical abstract(Fig. 1).

## Introduction

Malignant disorders represent a heterogeneous group of pathologies, being one of the major causes of morbidity and mortality worldwide (Kocarnik et al., 2022[64]); while in the context of overall mortality (0-X years), cancer is second major cause of death (with notable contributions due to lung, breast, colorectal, prostate, and liver cancers), they are the leading causes of mortality among the 18-64-year-old population, contributing substantially to years of potential life lost (YPLL) (Bray et al., 2024[17]). According to the estimates of the World Health Organization (WHO), there were ~20 million incident cases of cancer, with ~9.7 death arising from malignant disorders in 2020; furthermore, the burden of cancers is expected to grow by >70 % in the next 30 years, mediated by - among other things - global demographic changes, lifestyle characteristics and environmental exposures - which may strain national healthcare services past the breaking point (Jemal et al., 2011[57]). For example, in the Organisation for Economic Co-operation and Development (OECD) countries alone, cancer has been admitted to add ~450 billion euros of excess costs to the healthcare system annually (OECD, 2024[88]). Notwithstanding major advances in screening, biomarker-based molecular stratification of patients and novel treatment strategies (both pharmacological interventions and other modalities, such as precision surgery or radiotherapy), diseases are often still identified in advanced stages, often resulting in poor prognosis (WHO, 2002[133]). Conventional anticancer treatments - while effective in certain contexts - may often be hindered by limited cancer/healthy cell selectivity, and substantial, treatment-limiting toxicities, which may lead to substandard quality of life (QoL) (Zafar et al., 2025[142]). Furthermore, difficulties in oncological treatments are further compounded by tumor heterogeneity, the development of anticancer drug resistance, and the intricate interplay of signaling pathways affecting cell proliferation (Khan et al., 2024[61]). In these circumstances, targeted anticancer treatments, including kinase inhibitors (KIs), have become crucial tools to disrupt the specific signaling pathways that are responsible for cancer growth following malignant transformation.

Protein kinases (PKs) are enzymes, which play a critical role in regulating cellular signaling pathways that control cell proliferation, survival, processes of differentiation, programmed cell death (apoptosis) and autophagy (Dummler et al., 2009[28]; Fu et al., 2022[39]). Disruptions in the physiological functioning of these enzymes - either through mutations, their overexpression, or their abnormal activation is a characteristic hallmark of numerous cancers (e.g., BRAF in melanoma → leading to the constitutive activation of the MAP kinase pathway, HER2/ERBB2 overexpression in breast cancer, BCR-ABL fusion in chronic myeloid leukemia [CML] → leading to constitutive kinase activation), making PKs as attractive therapeutic targets for the development of novel anticancer drugs (Kannaiyan and Mahadevan, 2018[60]). Since the 2000s, KIs have revolutionized the care of oncological patients, by providing more selective and effective alternatives to conventional (cytotoxic) chemotherapy (Yegnasubramanian and Maitra, 2013[141]). Small-molecule KIs, particularly tyrosine kinase inhibitors (TKIs), have demonstrated remarkable clinical efficacy in the management of hematologic malignancies and solid tumors, significantly improving patient survival and QoL (Singh et al., 2022[111]). The ability of TKIs to target the specific molecular abnormalities to cancer cells has also advanced the paradigm of precision medicine in oncology, allowing treatment strategies to be tailored, based on genetic and molecular profiles determined in patients (Baldi et al., 2025[4]).

The U.S. Food and Drug Administration (FDA) plays a central role in ensuring that new anticancer therapeutics are both safe and effective before entering mainstream clinical use (FDA, 2025[36][37][32]). The approval of oncology drugs often follows a rigorous pathway that includes preclinical studies, phased clinical trials, and regulatory evaluation. In recent years, the FDA has increasingly adopted accelerated approval pathways, priority reviews, and designations of “breakthrough therapy” to expedite the availability of life-saving cancer treatments, especially those addressing unmet medical needs in specific indications (FDA, 2025[36][37][32]). This dynamic regulatory environment has facilitated the rapid introduction of novel KIs, often guided by strong biomarker-driven evidence (i.e. results from clinical trials which select patients based on the presence/absence of specific biological markers found in their blood or other tissues) (Ku et al., 2017[66]). As a result, FDA approvals not only set global standards for cancer therapeutics, but also drive innovation in drug development, providing incentives for pharmaceutical companies, and shaping clinical practice worldwide (FDA, 2025[36]).

The present year (2025) has so far witnessed a noteworthy amount of novel KI approvals by the FDA, reflecting substantial advances in oncology drug discovery and development. These newly approved agents expand the therapeutic arsenal of clinicians, offering improved efficacy, enhanced specificity, and in some cases, also better safety profiles compared with earlier generations of KIs (Roskoski, 2024[101]). Given the rapid pace of innovation in this field, a comprehensive evaluation of these novel agents is essential to understand their clinical impact, therapeutic positioning, and future potential in mainstream oncology (Kossakowski et al., 2025[65]). The present review aims to provide a current critical synthesis of the development, therapeutic potential, and clinical performance of KIs in oncology, highlighting both their transformative role and their inherent limitations in practice.

## Overview of the Role and Relevance of KIs in Anticancer Treatment

### Mechanism of action of KIs

**Figure 2:**
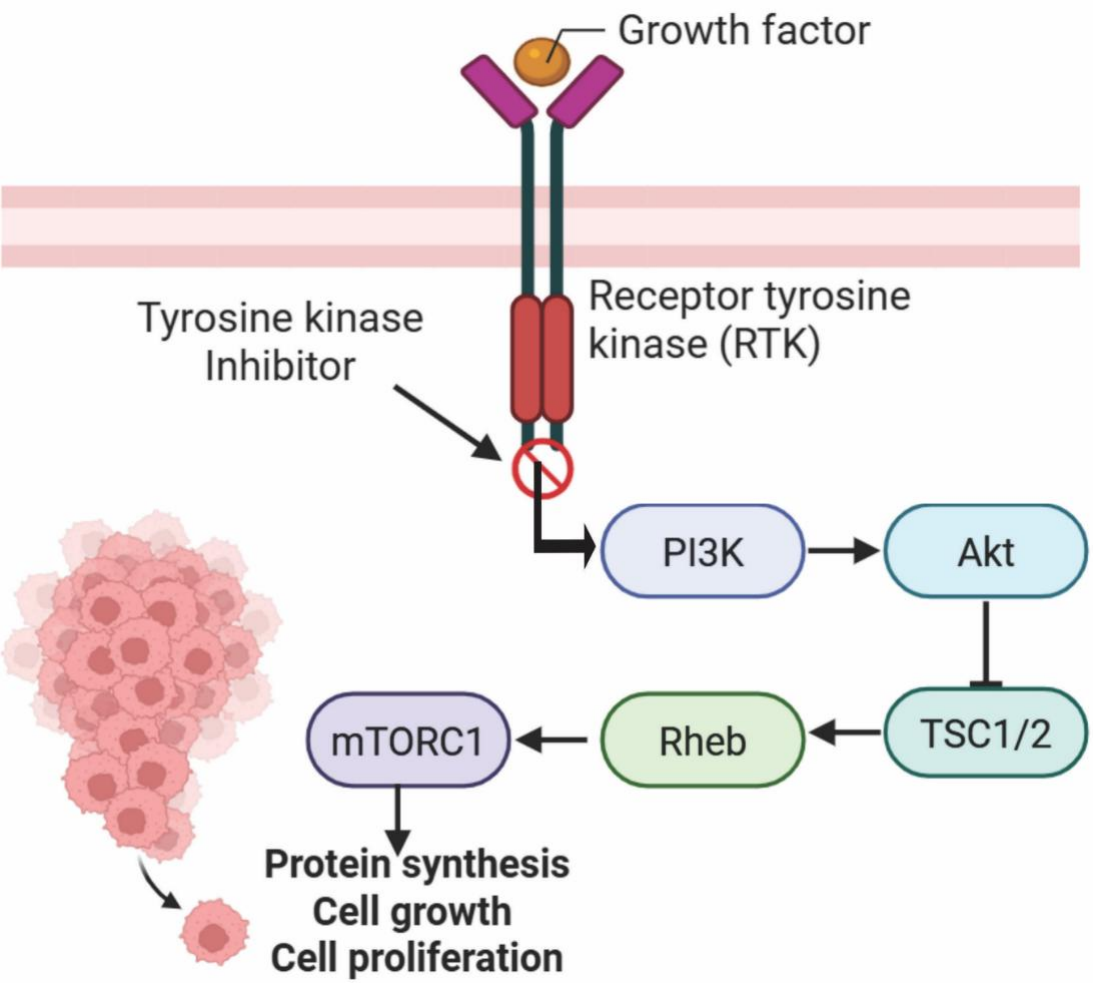
General mechanism of action of tyrosine kinase inhibitors (TKIs)

KIs act by targeting the dysregulated signaling pathways driving the process of malignant transformation and progression in tumor cells. Most small-molecule inhibitors function by competing with adenosine triphosphate (ATP) at the ATP-binding site of the PKs, thereby inhibiting phosphorylation events that are crucial for downstream signaling (e.g., imatinib, gefitinib, erlotinib) (Lui et al., 2022[77]). Furthermore, other agents exploit allosteric binding sites (e.g., trametinibm cobimetinib) or act irreversibly (such as ibrutinib, afatinib), providing greater specificity in their action. The general mechanism of action of TKIs is presented on Figure 2(Fig. 2). While this targeted approach has become a cornerstone of precision oncology, it is not without its demerits. Tumor heterogeneity, the emergence of secondary mutations, and the activation of adaptive regulatory pathways often lead to acquired therapeutic resistance, highlighting the incomplete durability of these agents (Mingione et al., 2023[84]; Wang et al., 2021[128]). Furthermore, the long-standing dichotomy between "on-target" and "off-target" effects (i.e. toxicities) continues to pose challenges in kinase inhibitor development. At the same time, while inhibition of the intended kinases underpins therapeutic efficacy (e.g., BCR-ABL inhibition by imatinib in chronic myeloid leukemia), off-target interactions may account for both beneficial effects in redundant pathways (such as VEGFR inhibition contributing to antiangiogenic activity) (Sayegh et al., 2023[105]; Shyam Sunder et al., 2023[110]) and adverse events that compromise tolerability (for instance, EGFR inhibition leading to dermatologic toxicities) (Shyam Sunder et al., 2023[110];^,^Wynn et al., 2011[137]). Thus, the mechanism of action of KPs, although conceptually straightforward, requires critical evaluation within the dynamic and adaptive tumor microenvironment *in vivo* (Gross et al., 2015[46]).

### Historical perspective of FDA approvals in the KI field

The approval of imatinib (Gleevec^®^; Novartis) in 2001 for the indication of CML marked a paradigm shift in clinical oncology, demonstrating that selective KIs could translate into unprecedented clinical outcomes. Since then, KIs have dominated the landscape of drug approvals in the oncology field, with >70 agents expected to enter the market by 2024 (Cohen et al., 2002[24]; Sacha, 2014[102]). Historically, the FDA has facilitated the development of TKIs through expedited pathways, particularly when supported by biomarker-driven evidence or robust clinical response rates, and improvements in refractory cancers (Latham et al., 2024[71]). However, this accelerated approval model is not without controversy: while it allows for earlier access to promising pharmaceuticals, post-marketing studies (i.e. post-authorization efficacy studies [PAES], evaluating the success of these drugs under real-world conditions) have sometimes failed to confirm survival benefits initially reported, raising concerns about the balance between regulatory flexibility and reliable clinical evidence in practice. A critical view of this trajectory highlights that - while KIs have undoubtedly revolutionized oncology - their approval process reflects a noteworthy tension between the urgency of addressing unmet clinical needs and the rigors of long-term efficacy and safety evaluations (Elbaz et al., 2024[29]; Roskoski, 2025[101]). 

### Current role in Standard of Care (SoC)

The most important characteristics of current anticancer drug classes - including their strengths, limitations and clinical indications - are summarized in Table 1(Tab. 1) (References in Table 1: Al Aboud et al., 2018[1]; Amjad, et al., 2020[2]; Choi and Lee, 2020[22]; Dai et al., 2024[25]; Kumar et al., 2024[68]; Kwilas et al., 2015[69]; Lovly and Shaw, 2014[76]; Mansoori et al., 2017[79]; Meng et al., 2024[81]; Bayat Mokhtari et al., 2017[12]; Nguyen et al., 2010[87]; Pan et al., 2009[92]; Shalata et al., 2024[107]; Shulman et al., 2014[109]; Tóth and Gaál, 2025[122]; Verschueren et al., 2025[126]; Zohourian and Brown, 2024[145]), highlighting the position of KIs in contemporary antitumor therapy. Today, KIs are firmly embedded in the SoC for multiple malignancies, including leukemias, lung cancer, breast cancer, renal cell carcinoma (RCC), and melanoma, respectively. They are often used as first-line therapies, particularly in biomarker-selected populations, and in many cases have replaced or significantly delayed the need for cytotoxic chemotherapy (Mukhopadhyay et al., 2012[86]). For example, imatinib revolutionized the treatment of CML, by targeting the BCR-ABL fusion protein, while erlotinib and osimertinib may also be considered standard therapies for non-small cell lung cancer (NSCLC) harboring epidermal growth factor receptor (*EGFR*) mutations (Araki et al., 2023[3]; Hurvitz and Kakkar, 2012[55]). In breast cancer, lapatinib has shown clinical utility in HER2-positive disease, whereas sunitinib and pazopanib remain important treatment options for advanced RCC (Cella and Beaumont; 2016[19], Puzanov et al., 2015[97]). In melanoma, vemurafenib and dabrafenib have substantially improved outcomes for patients with *BRAF V600E* mutations (Banzi et al., 2016[11]; Puzanov et al., 2015[97]).

Importantly, combination strategies, i.e. pairing KIs with immunotherapies, monoclonal antibodies (mAbs), or other targeted drugs have further expanded their clinical utility and improved durability of response in selected cancers (Miller et al., 2014[82]). Nevertheless, their integration into routine care is not without challenges: resistance mechanisms remain pervasive, often necessitating the sequential use of next-generation inhibitors (e.g., ponatinib in CML with the T315I mutation, or lorlatinib in ALK-positive NSCLC resistant to earlier ALK inhibitors) (Miller et al., 2014[82]; Sanford et al., 2015[103]). Additionally, economic considerations - particularly the high costs of long-term therapy - pose barriers to equitable access, especially in low- and middle-income countries (LMICs) (Hill et al., 2016[51]). Limited patient access and issues with marketing authorization of these next-generation drugs. Another critical limitation is that the majority of kinase inhibitors provide disease control rather than cure, underscoring the need for continued innovation (Feng et al., 2022[38]; Woudberg and Sinanovic, 2024[135]). In the following section, we detail the most current approvals of KIs by the FDA, highlighting their noteworthy attributes in the process. 

## FDA Novel Kinase Inhibitors Approved in 2025

### Zongertinib

Zongertinib (Hernexeos^®^; Boehringer Ingelheim Pharmaceuticals, Inc.) received FDA accelerated approval on August 8^th^, 2025, for the treatment of adults with unresectable or metastatic non-squamous NSCLC harboring HER2 (ERBB2) tyrosine kinase domain (TKD) activating mutations, following prior systemic therapy (FDA, 2025[35]). Patient selection is guided by an FDA-approved companion diagnostic, the *Oncomine Dx Target Test* (Fabbricatore, 2025[30]). This represents the first oral HER2-selective, irreversible tyrosine kinase inhibitor (TKI), which was approved for this molecularly defined NSCLC subset in the US (Heymach et al., 2025[50]). The approval is particularly noteworthy due to the fact that, while antibody drug conjugates (ADCs), such as trastuzumab deruxtecan (T-DXd) have shown efficacy in HER2-mutant NSCLC, their clinical use has been constrained by concerns of toxicity, including interstitial lung disease (ILD), and the need for intravenous (i.v.) administration (vs. the oral administration routes of KIs) (Li et al., 2022[72]). Thus, zongertinib provides a novel, convenient, and potentially safer oral option, although the indication remains limited to the post-systemic therapy setting under accelerated approval.

**Figure 3:**
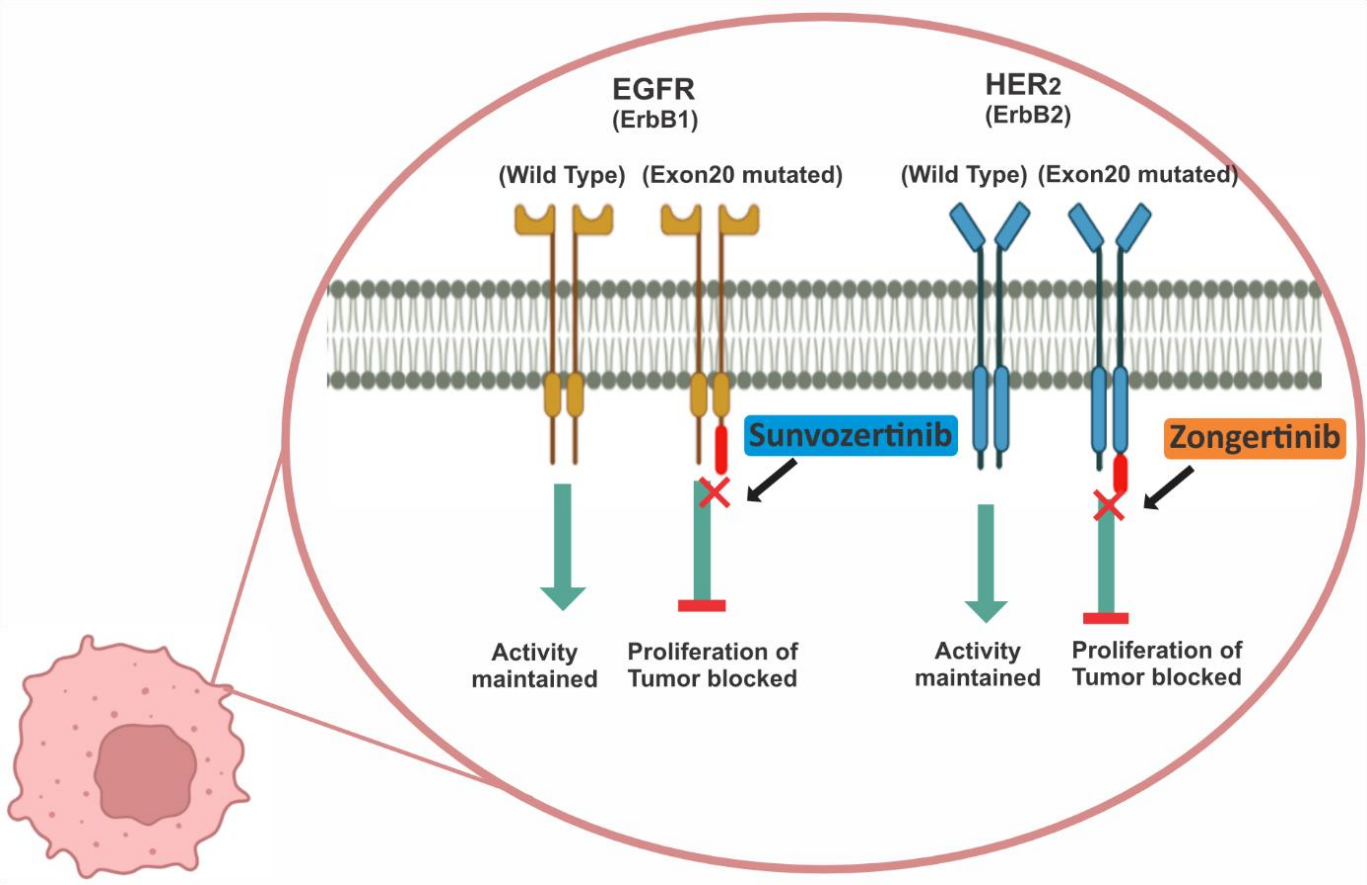
Visual representation of the mechanisms of action of sunvozertinib (Zegfrovy^®^) and zongertinib (Hernexeos®)

Mechanistically, zongertinib is an oral, irreversible HER2-selective inhibitor designed to covalently bind to the HER2 kinase domain, while sparing wild-type EGFR; the mechanism of action of zongertinib is shown on Figure 3(Fig. 3). This selectivity is clinically relevant, as it aims to mitigate the dermatologic (e.g., acneiform rash, dry skin, pruritus) and gastrointestinal toxicities (such as diarrhea and mucositis), typically associated with EGFR inhibition while maintaining potent anti-HER2 activity (Wilding et al., 2025[134]). The drug's development is rooted in the concept of oncogene addiction, exploiting HER2 mutations as a critical driver in NSCLCs (Son et al., 2022[113]). Data from preclinical studies also suggest a synergistic potential when combined with other targeted agents, including antibody-drug conjugates (ADCs) and KRAS^G12C inhibitors, though such strategies remain to be validated in clinical trials (Heymach et al., 2025[48]). 

The FDA approval was primarily supported by the Beamion LUNG-1 (NCT04886804) study, an open-label phase 1a/1b trial that evaluated zongertinib across multiple HER2 mutation cohorts. Among patients with HER2 TKD-activating mutations - who had received prior platinum-based (Pt-based) chemotherapy but no HER2-targeted therapy - the objective response rate (ORR) was 75 % (95 % CI: 63-83 %), with 58 % achieving a duration of response of six months or longer (Heymach et al., 2025[49]). In a more heavily pretreated cohort with prior HER2 ADC exposure, the ORR was reduced to 44 % (95 % CI: 29-61 %), with 27 % of patients sustaining responses beyond six months. Median progression-free survival (PFS) in the primary TKD-mutant group was reported at approximately 12.4 months, while the ADC-pretreated group achieved a median PFS of 6.8 months (Ismail et al., 2025[56]). These results underscore both the efficacy and durability of zongertinib, especially in patients who previously had limited therapeutic options available. Nonetheless, it must be emphasized that the data are derived from a single-arm, non-randomized trial with limited overall survival data, and confirmatory randomized studies, are essential to comprehensively establish its long-term clinical value.

The safety profile of zongertinib is consistent with its HER2-selective design. In the Beamion LUNG-1 trial, the most common treatment-related adverse events (AEs) were diarrhea and rash, which were generally low grade (grade 1-2) and manageable (Opdam et al., 2024[89]). Dose reductions were required in approximately 5 % of patients, while discontinuation due to adverse events occurred in only 3 % of cases. Serious toxicities, such as ILD, left ventricular dysfunction (LVD), and hepatotoxicity were rare in clinical trials, but they remain class-related concerns highlighted in the FDA prescribing information, necessitating ongoing monitoring (Wu et al., 2024[136]). Of particular note, unlike in the case of trastuzumab deruxtecan, no ILD cases were reported in the study, although broader clinical experience will be needed to confirm this favorable signal. The dosing regimen is weight-based and once daily (120 mg for patients <90 kg; 180 mg for those ≥90 kg), offering a convenient oral administration schedule that may improve adherence compared to intravenous therapies (Wu et al., 2024[136]).

Overall, zongertinib represents a first-in-class, HER2-selective oral TKI, that delivers compelling response rates and a favorable tolerability profile in a difficult-to-treat NSCLC population (Brazel et al., 2025[18]). The accelerated approval of this medication reflects both the unmet clinical need and the strength of early efficacy data, but ultimate role in the treatment paradigm will depend on the outcomes of ongoing confirmatory trials, for example, comparative effectiveness against HER2 ADCs, and its ability to maintain durable responses while preserving QoL. As such, zongertinib should be viewed as a notable advance in precision oncology, however, one that requires cautious optimism pending long-term clinical validation (Raggi et al., 2025[99]).

### Sunvozertinib

Sunvozertinib (DZD9008; Zegfrovy^®^; Dizal Pharma) is a next-generation oral EGFR TKI, that has been in development primarily for the treatment of NSCLC harboring EGFR exon 20 insertion mutations (EGFR Ex20Ins), a subset of EGFR alterations that are notoriously resistant to first- and second-generation EGFR inhibitors, leading to scarce treatment options. This makes it particularly relevant for patients with advanced NSCLC, who have limited targeted therapeutic options following conventional treatment failure (Wang et al., 2025[130]). The mechanism of action of sunvozertinib lies in its selective and potent inhibition of mutant EGFR Ex20Ins, while sparing wild-type EGFR to a greater degree than earlier inhibitors (see Figure 3(Fig. 3)). By binding to the ATP-binding site of the mutant receptor, it prevents downstream activation of proliferative and survival signaling pathways, particularly the MAPK and PI3K/AKT pathways, thereby reducing tumor cell growth and survival. This selective activity is critical, as exon 20 insertions induce structural changes that render tumors resistant to other antitumor drugs like osimertinib or afatinib, but remain susceptible to inhibition by sunvozertinib (Xu et al., 2025[139]).

Clinical trial data have shown encouraging efficacy outcomes. In a phase II study (WU-KONG6), sunvozertinib demonstrated an ORR of ~60 % in pretreated NSCLC patients with EGFR exon 20 insertions, with a median duration of response exceeding 9 months (Mitsudomi, 2025[85]). These results highlight a significant advance in targeted therapy for this patient population. The efficacy was consistent across different insertion subtypes, suggesting broad applicability within this genetic/biomarker-based patient subgroup (Wang et al., 2024[129]). The safety profile of sunvozertinib has generally been manageable, though not without notable AEs. The most common treatment-related side effects included diarrhea, rash, stomatitis, and decreased appetite, which are typical of EGFR-targeting agents (Liu et al., 2024[74]). Some patients also experienced paronychia and fatigue, in addition to other AEs Importantly, the incidence of high-grade toxicities appears lower than with certain other exon 20-directed therapies, and dose interruptions or reductions have generally allowed continued treatment. ILD, a rare but potentially serious toxicity associated with EGFR inhibitors, has been reported, but has been described relatively uncommonly in sunvozertinib-treated patients (Tucker, 2023[123]; Xie et al., 2025[138]).

### Avutometinib and defactinib

**Figure 4:**
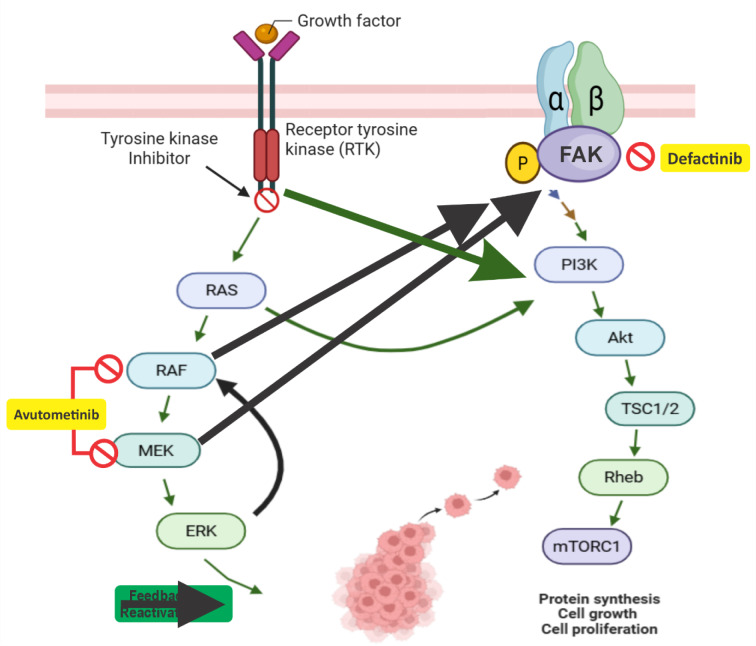
Visual representation of the mechanisms of action of avutometinib and defactinib combination (Avmapki^®^ Fakzynja^®^ Co-pack)

Avutometinib is an orally bioavailable inhibitor of the RAF-MEK axis (often described as a “RAF-MEK clamp”), which was developed to suppress the MAPK pathway signaling driven by upstream RAS/RAF mutations (Figure 4(Fig. 4)) (Blair, 2025[16]). In May 2025, the FDA granted accelerated approval to the co-packaged regimen of avutometinib plus defactinib (Avmapki^®^ Fakzynja^®^ Co-pack; Verastem Inc.) for adults with KRAS-mutated recurrent low-grade serous ovarian cancer (LGSOC), who have received prior systemic therapy, marking the first targeted approval for this rare ovarian cancer subtype, and reflecting the rationale of directly targeting MAPK pathway dependence in KRAS-mutant disease (Verdin, 2024[125]).The regulatory decision followed evidence from the RAMP-201 program (ENGOTov60/GOG3052) and supporting multi-center phase 2 data in this molecularly selected population (Banerjee et al., 2025[10]). Pharmacologically, avutometinib functions by locking RAF in a conformation that prevents productive RAF→MEK signaling, thereby reducing the downstream activation of the ERK pathway. This “clamp” mechanism differentiates it from the previous, “single-node” RAF or MEK inhibitors, because it aims to blunt adaptive reactivation of the MAPK pathway that typically limits the durability of classical MEK inhibitors (Stover et al., 2025[116]). The translational rationale is straightforward: KRAS-mutant LGSOC often relies on chronic MAPK signaling for proliferation and survival, so simultaneous and persistent suppression of RAF → MEK signaling should produce deeper and more durable tumor control than MEK inhibition alone. Preclinical models showed pathway suppression and antiproliferative activity that supported the subsequent clinical development of the drug (Pickard et al., 2025[96]). 

Clinical evidence supporting the activity of avutometinib (largely in combination with defactinib) comes from the RAMP-201 single-arm trial, and associated analyses. In the multi-center cohort reported to regulators and in recent peer-reviewed summaries, the combination achieved a confirmed ORR ~31 %, with a clinically meaningful disease control rate and responses observed in heavily pretreated patients who had limited prior options (Grisham et al., 2025[45]). These results were assessed by the FDA as sufficient for the accelerated approval pathway in a rare disease with unmet need, but the supporting data are derived from single-arm cohorts without randomized comparators; therefore, while response rates and durability signals are encouraging, the absence of randomized survival data limits definitive conclusions about the long-term clinical benefit and optimal sequencing versus other therapies (Beakes-Read et al., 2022[13]). The safety and tolerability of avutometinib has to be interpreted in the context of combination therapy; as a monotherapeutic agent, the AEs of avutometinib reflect other drugs acting through the inhibition of the MAPK pathway (i.e. rash, gastrointestinal effects, fatigue), but much of the safety profile in the approved indication is driven by combination exposure with defactinib. Reported toxicities in the initial reports included the expected dermatological and gastrointestinal effects, transient laboratory abnormalities, and treatment-emergent events requiring dose modification in a subset of patients. Given the accelerated approval pathway, post-marketing and confirmatory trial safety data (i.e. post-marketing safety studies [PASS]) will be critical for characterizing uncommon, but serious toxicities, and for understanding tolerability across broader, real-world patient populations, to ensure reliable risk-benefit analysis for clinicians (Lim et al., 2025[73]; Banerjee et al., 2025[9]).

Defactinib is an oral, ATP-competitive inhibitor of focal adhesion kinase (FAK) and the related kinase Pyk2. FAK is a non-receptor TK that integrates signals from integrins and growth factor receptors to regulate cell adhesion, migration, survival, and the tumor microenvironment; inhibiting FAK is therefore hypothesized to both impair the invasive properties of tumor cells, and to modulate stromal and immune components that promote tumor growth (Figure 4(Fig. 4)) (Banerjee et al., 2025[6]). Because FAK activity is implicated in KRAS-driven tumor biology and in stromal resistance mechanisms, the combination of a MAPK pathway clamp (avutometinib) with FAK inhibition (defactinib) was developed to specifically target both tumor-intrinsic signaling and pro-tumor stromal support, respectively (McNamara et al., 2024[80]). 

The clinical developmental pipeline of defactinib clinical development spans multiple tumor types and combination strategies. Earlier phase studies have established the tolerability and pharmacodynamic proof-of-mechanism of the molecule, while later trials explored combinations with MEK/RAF pathway inhibitors and with drug acting through immune checkpoint blockade. In the RAMP-201 program in KRAS-mutant LGSOC, defactinib paired with avutometinib contributed to the observed antitumor activity (confirmed ORR in the pivotal cohort and a favorable disease control signal) (Banerjee et al., 2025[9]). Beyond ovarian cancer, defactinib has shown modest single-agent activity in some other solid tumors, and has been investigated as a stromal/immune-modulating partner in mesothelioma, pancreatic cancer, and NF2-mutated tumors, respectively. Nonetheless, data suggest its primary value may be in combination regimens, rather than as a stand-alone cytotoxic agent. However, as with avutometinib, much of the strongest efficacy signal for defactinib comes from non-randomized studies, and randomized comparisons are still necessary to quantify incremental benefit (Banerjee et al., 2023[8]). The safety profile of defactinib has been described as generally manageable: across trials, the most common AEs have included fatigue, nausea, diarrhea, and reversible laboratory abnormalities. In combination regimens, overlapping toxicities with concomitant partner drugs (for example, MAPK pathway-related rash or gastrointestinal toxicity) have sometimes necessitated dose interruptions or reductions. Importantly, defactinib has not been associated with a characteristic life-threatening toxicity signal, but long-term combination use and larger post-approval experience are needed to define rarer AEs, interaction risks, and the tolerability of chronic administration in an often frail patient population (Gerber et al., 2020[42]; Seedor et al., 2024[106]).

Overall, the avutometinib-defactinib combination represents a mechanistically rational, biomarker-driven advance for KRAS-mutant recurrent LGSOCs. Avutometinib clamps RAF→MEK signaling, while defactinib targets FAK-mediated stromal/immune support. The combination produced objective responses in a disease with few targeted options, sufficient to secure accelerated approval. Nevertheless, the evidence base is dominated by single-arm data and relatively small cohorts; confirmatory randomized trials and expanded safety surveillance will be needed to establish the magnitude of clinical benefit, optimal sequencing, and long-term tolerability (McNamara et al., 2024[80]).

### Vimseltinib 

Vimseltinib (Romvimza^TM^; Deciphera Pharmaceuticals) is an oral, switch-control TKI approved by the FDA on February 14^th^, 2025, for the treatment of adult patients with symptomatic tenosynovial giant cell tumors (TGCTs), who are at risk of worsening functional limitation or severe morbidity, if surgical resection is performed. This approval follows the performance of a randomized, placebo-controlled trial, and positions vimseltinib as a non-surgical systemic option for a locally aggressive, often recurrent disease, in which surgery may lead to severe consequences or sequelae (FDA, 2025[34]). The mechanism of action of vimseltinib is centered on the selective inhibition of the activity of colony-stimulating factor-1 receptor (CSF1R). It is a “switch-control” inhibitor, that stabilizes an inactive conformation of CSF1R, thereby blocking CSF1-driven recruitment and survival signals for tumor-associated macrophages, and the CSF1-overexpressing neoplastic cells that characterize TGCT (Smith et al., 2021[112]). By the selective targeting of CSF1R , thereby sparing the physiological function of the other off-target kinases , vimseltinib aims to reduce prototypical inflammatory and macrophage-mediated stromal support, that drives TGCT growth and joint morbidity, underscoring its rationale in use and its tolerability profile compared with less selective KIs (Gelderblom et al., 2024[41]).

The MOTION (“Study of Vimseltinib for Tenosynovial Giant Cell Tumor”) phase-3 trial provided the principal evidence of efficacy for the approval of vimseltinib by the FDA. In the randomized, double-blind portion of the MOTION trial, vimseltinib produced a significant and clinically meaningful improvement in ORR at Week 25: 40 % in the vimseltinib arm versus 0 % in the placebo arm, respectively (difference 40 %; 95 % CI: 29-51 %; *p*<0.0001), by independent radiologic review using RECIST v1.1 (Bernthal et al., 2024[15]). Tumor-volume score (TVS) responses and measures of active range of motion also favored the use of vimseltinib, and the study met key secondary endpoints, including patient-reported outcomes (PROMs) related to everyday function and pain. These results are robust for a rare, locally aggressive neoplasm, and represent the first large randomized evidence that selective CSF1R inhibition may meaningfully decrease disease burden and improve function in TGCT (Gelderblom et al., 2024[40]). Safety data from the MOTION trial and the review by the FDA indicated that the AE profile of vimseltinib is generally manageable but active monitoring may be necessary. The most common AEs (≥20 %) through Week 25 included increased aspartate aminotransferase (AST), periorbital edema, fatigue, rash, increased cholesterol, peripheral and facial edema, neutropenia/leukopenia, pruritus, and increased alanine aminotransferase (ALT), respectively. Grade 3-4 events observed more frequently in the vimseltinib arm, which included elevations in creatine phosphokinase (CPK), hypertension, and select laboratory abnormalities; the label carries warnings to monitor liver function and other laboratory parameters and recommends dose interruptions or discontinuation in the case of significant toxicity. Overall, no new class-defining, life-threatening toxicity signal emerged during the MOTION trial, but the requirement for serial liver tests and vigilance for edema, cytopenias, and CPK elevations has been emphasized (Tap et al., 2024[120]). 

Critically, vimseltinib represents a well-justified, mechanism-based advance in the management of TGCT: the randomized MOTION data provide higher-quality evidence than many approvals in the context of rare tumors that rely solely on single-arm studies (Tap et al., 2024[121]). Nevertheless, some caveats remain: TGCT is a locally aggressive, non-metastatic disease, where the clinical aim is function preservation and symptom control, rather than prolongation of OS. Therefore, the long-term durability of response, impact on surgical rates, real-world tolerability, and QoL gains beyond the trial period will determine the ultimate clinical value of vimseltinib. In addition, as vimseltinib is taken intermittently (with recommended dosing at 30 mg twice weekly) and requires laboratory monitoring, practical implementation and adherence considerations will influence outcomes outside clinical trial settings. Continued post-marketing surveillance (PASS and PAES studies) and longer-term cohort data will therefore be important to confirm both the sustained benefit and the absence of rare, serious toxicities of its use (Qadri et al., 2025[98]; Wagner et al., 2024[127]). 

### Mirdametinib

Mirdametinib (Gomekli^®^; SpringWorks Therapeutics Inc.) is an orally bioavailable, selective inhibitor of MEK1/2, which was developed to target cancers driven by mutations in the RAS/MAPK signaling cascade. The primary indication of the drug has been in the context of rare tumors, such as neurofibromatosis type 1 (NF1)-associated plexiform neurofibromas, where treatment options are extremely scarce; however, the drug is also being investigated towards the use in solid tumors with MAPK pathway alterations (Hoy, 2025[52]). The drug acts by inhibiting MEK1/2, thereby blocking ERK phosphorylation and downstream oncogenic signaling, which is critical in regulating cell proliferation, differentiation, and survival in tumors harboring RAS or RAF mutations (Hao et al., 2023[47]). Clinical trial data, particularly from the ReNeu Phase II study, demonstrated that mirdametinib achieved clinically meaningful tumor shrinkage and symptom improvement in patients with NF1-related plexiform neurofibromas, with response rates >40 %. Importantly, patients also reported reductions in pain and improvements in QoL, highlighting the dual efficacy of this treatment in both tumor control and symptom relief. Ongoing studies are evaluating its role in a broader range of MAPK-driven malignancies, such as low-grade gliomas and other RAS-mutant solid tumors, further broadening its therapeutic relevance (FDA, 2025[33]; SpringWorks Therapeutics, 2023[114], 2025[115]). The safety profile of mirdametinib has been described as generally manageable, with the most common AEs being acneiform rash, diarrhea, nausea, fatigue, and peripheral edema, consistent with AEs seen in other MEK inhibitors. Dose interruptions or reductions are may be required due to dermatologic or gastrointestinal toxicity, though severe events remain relatively uncommon (Shin et al., 2024[108]). Overall, mirdametinib represents a promising precision therapy for patients with NF1, and potentially other cancers in the future, which are dependent on MAPK pathway signaling, balancing clinical benefit with an acceptable tolerability profile (Weiss et al., 2021[132]).

## Clinical Efficacy of Newly Approved Drugs Versus Prior KIs

The FDA approvals seen in the KIs sphere in 2025 represent a mix of paradigm-shifting, niche-targeted, and incremental advances in clinical care of oncological patients. Zongertinib and sunvozertinib both address previously refractory, mutation-defined NSCLC subgroups (i.e. HER2 TKD mutations and EGFR exon-20 insertions, respectively) and produced high-level ORRs and clinically meaningful median progression-free survival rates as outcomes in their respective single-arm trials, that clearly exceed historic activity from conventional chemotherapy and older, non-selective TKI drugs in these populations (Xu et al., 2025[139]; Zeng et al., 2021[143]). As these cohorts previously relied on off-label TKI use, chemotherapy, or intravenous ADCs with different toxicity/administration profiles, the availability of novel, oral targeted options would meaningfully broaden therapeutic choices for clinicians and patients alike (Oztosun et al., 2025[91]). The avutometinib+defactinib approval for KRAS-mutant recurrent LGSOCis the first targeted approval for this rare indication, which have shown ORs in a disease with few effective systemic options available; however, the evidence derives largely from non-randomized cohorts, so its comparative advantage versus prior practice and SoC (often hormonal therapy or chemotherapy) is promising, but provisional at best (Blair, 2025[16]). Mirdametinib and vimseltinib are approvals in rare, non-metastatic or benign but morbid disease settings (NF1-PN for mirdametinib; symptomatic TGCT for vimseltinib, respectively) where prior systemic options were limited; randomized or historically controlled data demonstrate a clear benefit in function and tumor shrinkage for vimseltinib (in a randomized, phase-3 trial), and meaningful response/symptom benefit for mirdametinib, making both more definitively practice-changing within their narrow indications (FDA, 2025[33][36][37][32]). Thus, these approvals range from high-impact across previously untreatable molecular subgroups (i.e. sunvozertinib, zongertinib) to important first-in-class options in rare diseases (i.e. avutometinib+defactinib, vimseltinib, mirdametinib), however, the strength of evidence varies (randomized evidence strongest for vimseltinib; single-arm evidence for several others), warranting additional confirmation from the triangulation of future evidence (Lokaj, 2023[75]; Wang et al., 2022[131]).

## Safety and Tolerability Newly Approved Drugs

A consistent theme across these novel drug approvals was seen, i.e. the engineering for selectivity to widen the therapeutic window of drugs. Zongertinib is HER2-selective (designed to spare wild-type EGFR), and sunvozertinib is mutant-selective for EGFR exon-20 insertions, with relatively lower wild-type EGFR activity. Both strategies aimed to reduce class-typical dermatologic and gastrointestinal toxicity, commonly seen during the use of these medicines (Son et al., 2022[113], Wilding et al., 2025[134]). Clinical trials reported mainly low-to-moderate grade diarrhea, rash, and GI effects that were manageable with dose modification. However, class-relevant serious risks (such as hepatotoxicity, cardiac effects, ILD/pneumonitis) are included in labels or precautions, and require subsequent monitoring (Brazel et al., 2025[18]; Yang et al., 2024[140]). The toxicity profile of the co-pack of avutometinib+defactinib is mediated by the MAPK pathway and FAK inhibition (i.e. rash, GI toxicities, fatigue, laboratory abnormalities) and may often require dose reductions when used in combination (Banerjee et al., 2025[6][9]). Randomized data on vimseltinib showed predictable laboratory alternation and edema signals (liver enzyme elevations, edema, neutropenia), and a tolerability profile that was determined as acceptable in the MOTION trial with monitoring recommendations; as the MOTION was randomized, clinicians may more confidently weigh benefit versus harms (Tap et al., 2024[120]). The AE profile of mirdametinib mirrors that of other MEK inhibitors (acneiform rash, diarrhea, fatigue), but was considered manageable in NF1 studies. Overall, while selective targeting has reduced some of the off-target toxicities compared with older, less selective TKIs, none of the newly approved agents are toxicity-free: monitoring (liver tests, cardiac assessment, ILD vigilance) and active management of gastrointestinal/dermatological effects remain central to their safe use. Comparative tolerability therefore favors the more selective designs, but still demands real-world pharmacovigilance studies in the future (FDA, 2025[33][35][36][37][32], SpringWorks Therapeutics, 2023[114], 2025[115]). 

## Impact on Treatment Guidelines and Standards of Care Following the Approval of Novel Drugs

The approvals of these novel targeted anticancer drugs by the FDA will drive relatively rapid updates in guideline algorithms most obviously for molecularly-defined NSCLC subsets. of the use of accelerated pathways by the regulatory authority largely reflects the high observed unmet need, and may lead to early adoption of these medicines in practice, with guideline committees likely to add sunvozertinib and zongertinib as recommended options for post-Pt (and biomarker-confirmed) disease, while stipulating the need for confirmatory data in the future (Jeon et al., 2025[58]). For rare diseases (such as KRAS-mutant LGSOC, TGCT, NF1-PN, respectively), the new agents will likely become SoC systemic options, where surgical interventions or earlier systemic choices were inadequate; for vimseltinib, the randomized, phase-3 evidence is strong enough to achieve changing practices rapidly in TGCT (Huang et al., 2025[54]). However, guideline panels should balance enthusiasm with caution: many of the mentioned approvals in 2025 rely solely on single-arm data, therefore implementation of guideline recommendations will often be conditional, and accompanied by calls for confirmatory randomized evidence and long-term safety surveillance (PDQ Cancer Information Summaries, 2025[93]). Additionally, real-world access (including the costs, technical capability and availability of diagnostic testing) will influence their implementation and integration into guidelines globally. High prices and the precondition for companion diagnostics may delay widespread adoption in low-resource settings, thereby contributing to disparities worldwide. As an indirect consequence, these approvals may accelerate the uptake of molecular testing modalities (e.g., expanded use of next-generation sequencing [NGS] panels to detect HER2 TKD and EGFR exon-20 insertions), which presents as a necessary infrastructural shift to allow for guideline-concordant care (Jeon et al., 2025[58]; Owen et al., 2024[90]). 

## Translational and Clinical Implications, Future Challenges

The recent wave of novel oncology drug approvals in 2025 underscores the accelerating shift toward biomarker-driven precision medicine in this field, where therapeutic efficacy is closely tied to molecular profiling of cancers, and by extension, the patients who may receive these treatments (Kudek et al., 2025[67]). For instance, sunvozertinib has demonstrated response rates ~60 % in patients with EGFR exon 20 insertion-mutated NSCLC, a population where resistance to first- and second-generation EGFR inhibitors is consistently being described (Xie et al., 2025[139]). Similarly, avutometinib combined with defactinib produced ORR~45-50 % in KRAS-mutant LGSOC, which may be considered a meaningful improvement compared with the results from traditional chemotherapy, which rarely exceeds ORRs over 10-15 %. These results highlight that the efficacy of these agents is not only superior to prior KIs, but also marks a substantial advance in previously treatment-recalcitrant cancers (Grisham et al., 2024[44]). However, the durability of treatment response remains an area of concern; for example, median progression-free survival with sunvozertinib was reported at around 8 months, emphasizing the potential for the development of resistance occurring at a rapid rate, and the need for rational combination strategies (Zhou et al., 2025[144]).

Safety and tolerability comparisons further highlighted the delicate balance between efficacy and long-term patient management: many of these KIs share overlapping toxicities, such as rash, diarrhea, and hepatotoxicity, yet they may differ in severity and frequency. Sunvozertinib and zongertinib, for example, were associated with lower rates of dose-limiting pneumonitis compared with earlier EGFR inhibitors, but gastrointestinal side effects were relatively common, with grade ≥3 diarrhea observed in about 15-20 % of patients (Mina et al., 2025[83]; Sun et al., 2024[117]). In contrast, avutometinib/defactinib combinations showed a higher incidence of hematological toxicities (neutropenia, anemia), though they were manageable with supportive care (Banerjee, 2023[7]). Vimseltinib, designed for the treatment of TGCT, demonstrated a more favorable tolerability profile, with most AEs limited to low-grade edema and fatigue, highlighting a more patient-friendly spectrum of toxicity (Kisielewska and Rutkowski, 2025[63]). Collectively, the presently available data suggests incremental improvements in tolerability, though the challenge remains in cumulative toxicity with prolonged use, especially in chronic malignancies requiring continuous therapy (Chan et al., 2025[20]).

From the standpoint clinical guideline and practice development, these approvals are poised to reshape SoC care in narrow, molecularly defined populations. Sunvozertinib and zongertinib are likely to be incorporated into National Comprehensive Cancer Network (NCCN) and European Society for Medical Oncology (ESMO) guidelines as first- or second-line options for EGFR exon 20 NSCLC, where therapeutic choices were previously limited to amivantamab (Rybrevant^®^) or mobocertinib (Exkivity^®^) respectively, both of which had ORRs <40 %. Similarly, avutometinib/defactinib is positioned to become the preferred systemic option for KRAS-mutant LGSOC, shifting treatment away from empiric chemotherapy (Brazel et al., 2025[18]; Mitsudomi, 2025[85]; Wang et al., 2022[131]). The integration of vimseltinib into treatment algorithms for TGCT is also notable, as it represents the first rationally designed CSF1R inhibitor with durable disease control, potentially reducing the reliance on repeated surgical interventions (Killock, 2024[62]). Overall, the clinical and translational implications of these approvals reflect a notable shift: oncology is moving from broadly cytotoxic approaches to biomarker-informed, mechanism-based interventions that not only prolong survival, but also improve the quality of care and QoL for previously underserved patient populations.

The therapeutic benefits of novel KIs are tempered by the inevitable development of resistance, often mediated via the occurrence of secondary mutations or activation of alternative signaling pathways influencing the cell cycle. While multiple clinical trials are underway to expand the use of the above mentioned drugs, evidence on long-term survival and safety is still scarce, requiring additional evidence. Combination approaches with immunotherapy or chemotherapy hold promise for enhancing efficacy and delaying resistance development. Nonetheless, the appearance of the mentioned novel KIs may also introduce additional challenges of added toxicity, higher therapeutic costs, and the need for precise biomarker-driven patient selection to reach the most added benefit vs. previously available treatment options (Hu and Dignam, 2019[53]). In this context, additional data on PROMs associated with their administration - including capturing patient and caregiver perspectives on symptom burden, functionality and QoL - may further emphasize the benefits of these new approvals, providing a more convincing reason to amend regulatory (and reimbursement mechanisms) in their favor (Balitsky et al., 2024[5]; Churruca et al., 2021[23]; Malandrini et al., 2024[78]). However, especially in the case of LMIC regions, where the burden of malignant diseases is rising the most rapidly access to innovative therapies, such as the drugs discussed in this review is severely delayed or hindered, presenting as barriers to entry (Dare et al., 2021[26]). Therefore, ensuring more equitable access to the state-of-the-art treatments available would underscore their broader societal and clinical value on a global scale. Of course, from the perspectives of health insurance agencies and organizations deciding on reimbursement strategies, the appropriate cost-effectiveness analyses will need to be carried out, to put the innovations into the context of health economics and outcomes research (Davidoff et al., 2022[27]). 

## Patient-Reported Outcome Measures (PROMs) and Quality of Life (QoL) Considerations in Contemporary Anticancer Treatments

An increasingly important aspect of evaluating TKI-based cancer therapy lies in the integration of patient-reported outcomes (PROs), which provide direct insight into how treatment affects patients' daily functioning (and the corresponding burden - or lack thereof - on their caregivers), changes in their symptoms, and overall QoL (Perry et al., 2024[95]). Unlike traditional clinical endpoints, such as progression-free survival or OS, PROMs capture the real-world tolerability and humanistic value of therapeutic interventions (Bellino and La Salvia, 2024[14]). Several studies have shown that KIs, such as osimertinib and alectinib in NSCLC, are associated with improved health-related quality of life (HRQoL) scores compared to cytotoxic chemotherapy, largely due to reduced fatigue, nausea, and hematological toxicity (Chang et al., 2024[21]; Jovanoski et al., 2025[59], Santarpia et al., 2017[104]). However, chronic low-grade AEs, such as diarrhea, rash, or hepatotoxicity may accumulate over prolonged use, influencing adherence and psychological well-being (Chang et al., 2024[21]). Importantly, integrating validated PROM instruments (e.g., EORTC QLQ-C30, FACT-G instruments) into clinical trials and post-marketing surveillance of newly FDA approved KIs enables a more holistic assessment of therapeutic value (Lai-Kwon et al., 2024[70]). Development of future KIs should, therefore, adopt a dual-evaluation model, balancing survival benefits with sustained QoL, to ensure that advances in molecular oncology translate into meaningful improvements from the patient's perspective, underscoring the benefits of the treatment for reimbursement agencies as well (Pe et al., 2025[94]).

## Access and Affordability of Novel KIs

Access and affordability remain major challenges in the global implementation of KI-based cancer therapy, and their wider adoption in local clinical guidelines (Hill et al., 2016[51]). While these agents have revolutionized the management of multiple malignancies discussed above, their high cost and limited reimbursement frameworks have created significant disparities between high-income and LMICs (Roskoski, 2024[100]). For instance, the monthly cost of a single KI, such as osimertinib or lorlatinib, may exceed US$10,000-15,000, rendering long-term treatment unsustainable for most patients without a comprehensive insurance plan or national subsidy programs offered for relevant pharmaceutical companies to urge their presence in national markets (Woudberg and Sinanovic, 2024[135]). Moreover, delayed regulatory approvals, lack of generic alternatives, and fragmented supply chains of newly FDA-approved KIs further restrict patient access in resource-scarce settings (Latham et al., 2024[71]; Lui et al., 2022[77]).

Even within developed healthcare systems, cost-related nonadherence has emerged as a critical issue, leading to suboptimal therapeutic outcomes despite availability of these drugs, which may also be affected by their AE profiles (Tan et al., 2021[119]). The integration of health economics and outcomes-based analyses even early as clinical trial design is therefore essential to evaluate not only efficacy but also cost-effectiveness and real-world value (Goranova-Marinova et al., 2024[43]). Expanding access to generic and biosimilar alternatives (when relevant), establishing international price negotiations, and incorporating these drugs into essential medicines lists (e.g., the WHO Essential Medicine Initiative) are key policy priorities (Talon et al., 2021[118]). Ensuring equitable access to KIs is not merely an economic consideration, but a fundamental component of global cancer care equity, determining whether scientific innovation translates into population-level survival gains in cancer (Vaez-Gharamaleki and Hosseini, 2024[124]).

## Conclusion

The tendencies observed through the recently approved KIs point toward the movement of oncology towards precision medicine and ever finer molecular stratification, the preference towards orally-bioavailable, mutation-selective options for patient populations who previously had limited treatment prospects, and the emergence of randomized evidence in the context of selected rare diseases. The clinical promise of the novel KIs is substantial, but heterogeneity in the quality of evidence (randomized vs single-arm), lingering safety monitoring needs, and the necessary prerequisites of these treatments (diagnostic capacities, market access) mean that their ultimate, durable impact on survival and global standards of care will depend on confirmatory trials, post-marketing safety data, and their equitable implementation into routine clinical care.

## Declaration

### Acknowledgments

The authors are thankful for the support of the Study Group for Dental Research Methodology and Health Sciences, University of Szeged.

### Funding

The research received no external funding.

### Data availability statement

All data generated during the study are presented in this paper.

### Conflict of interest

The authors declare no conflicts of interest, monetary or otherwise. The authors alone are responsible for the content and writing of this article.

### Artificial Intelligence (AI ) - Assisted Technology

We acknowledge the use of ChatGPT [https://chat.openai.com/] in helping us to review English writing at the final stage of preparing the manuscript.

### Author contributions

MA; Conceptualization, Supervision, Literature search, Data curation, Writing - original draft, SHAS; Literature search, Data validation, Writing - review & editing, MG (Márió Gajdács); Critical revision of the manuscript, Writing - review & editing, Project administration, MJT; Literature review, Visualization, Writing - review & editing. All authors have read and approved the final version of the manuscript.

## Figures and Tables

**Table 1:**
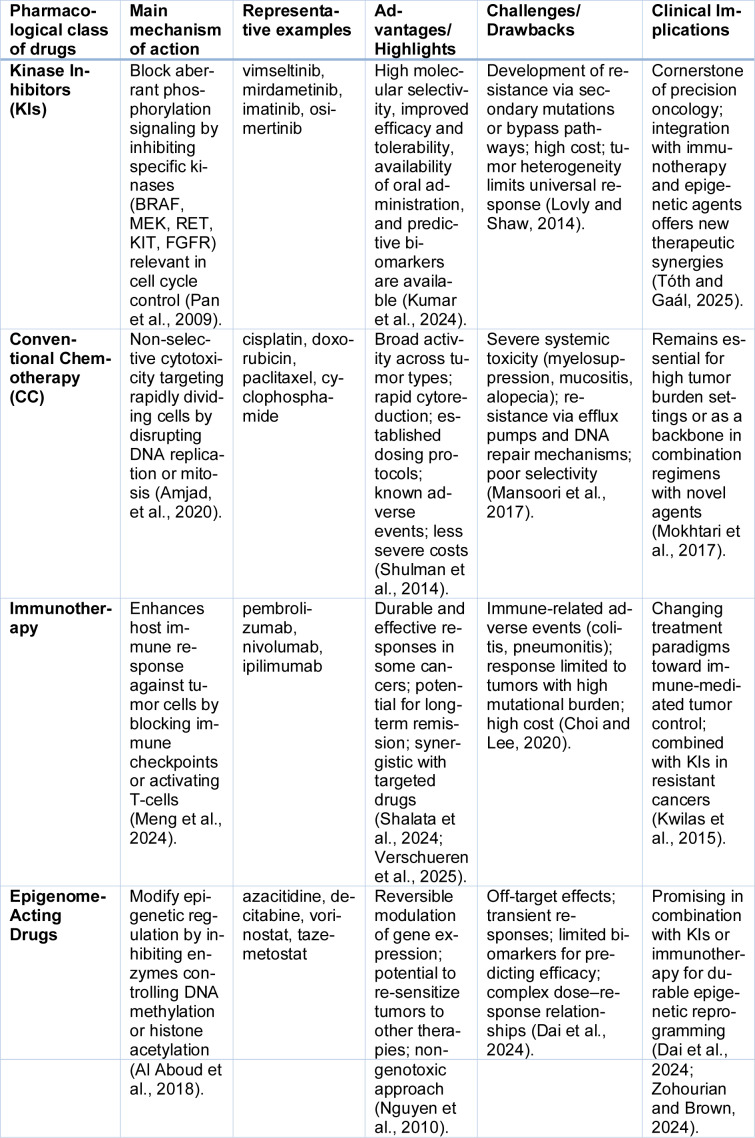
Comparative overview of major classes of clinically-relevant anticancer therapies, with an emphasis on their mechanism of action, advantages and limitations, and clinical aspects in contemporary oncology

**Figure 1:**
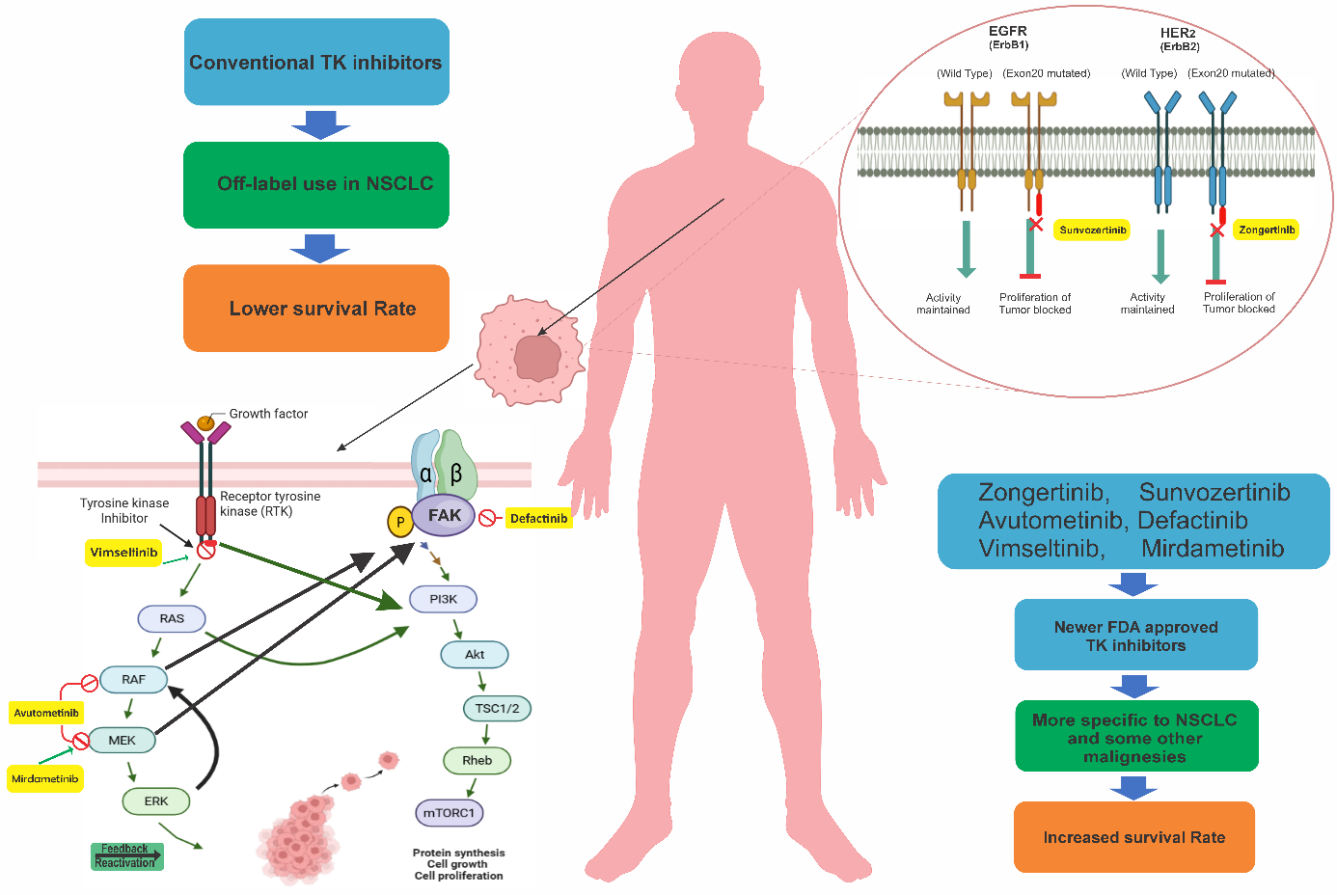
Graphical abstract

## References

[1] Al Aboud NM, Tupper C, Jialal I (2018). Genetics, epigenetic mechanism. https://www.ncbi.nlm.nih.gov/books/NBK532999/.

[2] Amjad MT, Chidharla A, Kasi A (2020). Cancer Chemotherapy. https://pubmed.ncbi.nlm.nih.gov/33232037/.

[3] Araki T, Kanda S, Horinouchi H, Ohe Y (2023). Current treatment strategies for EGFR-mutated non-small cell lung cancer: from first line to beyond osimertinib resistance. Jpn J Clin Oncol.

[4] Baldi S, Long N, Ma S, Liu L, Al-Danakh A, Yang Q (2025). Advancements in Protein Kinase Inhibitors: From Discovery to Clinical Applications. Research (Wash D C).

[5] Balitsky AK, Rayner D, Britto J, Lionel AC, Ginsberg L, Cho W (2024). Patient-Reported Outcome Measures in Cancer Care: An Updated Systematic Review and Meta-Analysis. JAMA Network Open.

[6] Banerjee S, Krebs MG, Greystoke A, Garces AI, Perez VS, Terbuch A (2025). Defactinib with avutometinib in patients with solid tumors: the phase 1 FRAME trial. Nature Medicine.

[7] Banerjee SN (2023). Avutometinib/Defactinib May be a New SOC in Low-Grade Serous Ovarian Cancer. Cancer Network.

[8] Banerjee SN, Ring KL, Nieuwenhuysen EV, Fabbro M, Aghajanian C, Oaknin A (2023). Initial efficacy and safety results from ENGOT-ov60/GOG-3052/RAMP 201: A phase 2 study of avutometinib (VS-6766) ± defactinib in recurrent low-grade serous ovarian cancer (LGSOC). J Clin Oncol.

[9] Banerjee SN, Van Nieuwenhuysen E, Aghajanian C, D'Hondt V, Monk BJ, Clamp A (2025). Efficacy and Safety of Avutometinib ± Defactinib in Recurrent Low-Grade Serous Ovarian Cancer: Primary Analysis of ENGOT-OV60/GOG-3052/RAMP 201. J Clin Oncol.

[10] Banerjee SN, Van Nieuwenhuysen E, Aghajanian C, D'Hondt V, Monk BJ, Clamp A (2025). Efficacy and Safety of Avutometinib ± Defactinib in Recurrent Low-Grade Serous Ovarian Cancer: Primary Analysis of ENGOT-OV60/GOG-3052/RAMP 201. J Clin Oncol. 2025b;43(25):2782-2792. doi: 10.1200/JCO-25-00112. Erratum in: J Clin Oncol.

[11] Banzi M, De Blasio S, Lallas A, Longo C, Moscarella E, Alfano R (2016). Dabrafenib: a new opportunity for the treatment of BRAF V600-positive melanoma. Onco Targets Ther.

[12] Bayat Mokhtari R, Homayouni TS, Baluch N, Morgatskaya E, Kumar S, Das B (2017). Combination therapy in combating cancer. Oncotarget.

[13] Beakes-Read G, Neisser M, Frey P, Guarducci M (2022). Analysis of FDA's Accelerated Approval Program Performance December 1992-December 2021. Ther Innov Regul Sci.

[14] Bellino S, La Salvia A (2024). The Importance of Patient Reported Outcomes in Oncology Clinical Trials and Clinical Practice to Inform Regulatory and Healthcare Decision-Making. Drugs R D.

[15] Bernthal NM, Stern S, Blay JY (2024). Vimseltinib versus a placebo in patients with tenosynovial giant cell tumor: a plain language summary of the MOTION phase 3 trial. Future Oncol.

[16] Blair HA (2025). Avutometinib and Defactinib: First Approval. Drugs.

[17] Bray F, Laversanne M, Sung H, Ferlay J, Siegel RL, Soerjomataram I (2024). Global cancer statistics 2022: GLOBOCAN estimates of incidence and mortality worldwide for 36 cancers in 185 countries. CA: a cancer journal for clinicians.

[18] Brazel D, Park CJ, Nagasaka M (2025). The development of Zongertinib for HER2-mutant NSCLC. Critical Reviews in Oncology/Hematology.

[19] Cella D, Beaumont JL (2016). Pazopanib in the treatment of advanced renal cell carcinoma. Ther Adv Urol.

[20] Chan SPY, Rashid MBMA, Lim JJ, Goh JJN, Wong WY, Hooi L (2025). Functional combinatorial precision medicine for predicting and optimizing soft tissue sarcoma treatments. npj Precision Oncology.

[21] Chang ZH, Zhu TF, Ou W, Jiang H, Wang SY (2024). A real-world retrospective study to assess efficacy and safety of alectinib as adjuvant therapy in IB-IIIB NSCLC patients harboring ALK rearrangement. Front Oncol.

[22] Choi J, Lee SY (2020). Clinical Characteristics and Treatment of Immune-Related Adverse Events of Immune Checkpoint Inhibitors. Immune Netw.

[23] Churruca K, Pomare C, Ellis LA, Long JC, Henderson SB, Murphy LED (2021). Patient-reported outcome measures (PROMs): A review of generic and condition-specific measures and a discussion of trends and issues. Health Expect.

[24] Cohen MH, Williams G, Johnson JR, Duan J, Gobburu J, Rahman A (2002). Approval summary for imatinib mesylate capsules in the treatment of chronic myelogenous leukemia. Clin Cancer Res.

[25] Dai W, Qiao X, Fang Y, Guo R, Bai P, Liu S (2024). Epigenetics-targeted drugs: current paradigms and future challenges. Signal Transduction and Targeted Therapy.

[26] Dare AJ, Knapp GC, Romanoff A, Olasehinde O, Famurewa OC, Komolafe AO (2021). High-burden Cancers in Middle-income Countries: A Review of Prevention and Early Detection Strategies Targeting At-risk Populations. Cancer Prev Res (Phila).

[27] Davidoff AJ, Akif K, Halpern MT (2022). Research on the economics of cancer-related health care: an overview of the review literature. JNCI Monographs.

[28] Dummler B, Ohshiro K, Kumar R, Field J (2009). Pak protein kinases and their role in cancer. Cancer Metastasis Rev.

[29] Elbaz J, Haslam A, Prasad V (2024). An empirical analysis of overall survival in drug approvals by the US FDA (2006–2023). Cancer Medicine.

[30] Fabbricatore R, Conroy R (2025). Cancer Network. FDA Approves NGS-Based Companion Diagnostic for Zongertinib in NSCLC. https://www.cancernetwork.com/view/fda-approves-ngs-based-companion-diagnostic-for-zongertinib-in-nsclc?utm_source=chatgpt.com.

[31] FDA, Food and Drug Administration (2018). Center for Drug Evaluation and Research, Application Number. 761094Orig1s000, Clinical Review. https://www.accessdata.fda.gov/drugsatfda_docs/nda/2018/761094Orig1s000MedR.pdf.

[32] FDA, Food and Drug Administration (2025). FDA Approvals in Oncology: April-June. https://www.aacr.org/blog/2025/07/01/fda-approvals-in-oncology-april-june-2025/.

[33] FDA, Food and Drug Administration (2025). FDA approves mirdametinib for adult and pediatric patients with neurofibromatosis type 1 who have symptomatic plexiform neurofibromas not amenable to complete resection. https://www.fda.gov/drugs/resources-information-approved-drugs/fda-approves-mirdametinib-adult-and-pediatric-patients-neurofibromatosis-type-1-who-have-symptomatic.

[34] FDA, Food and Drug Administration (2025). FDA approves vimseltinib for symptomatic tenosynovial giant cell tumor. https://www.fda.gov/drugs/resources-information-approved-drugs/fda-approves-vimseltinib-symptomatic-tenosynovial-giant-cell-tumor.

[35] FDA, Food and Drug Administration (2025). FDA grants accelerated approval to zongertinib for non-squamous NSCLC with HER2 TKD activating mutations. https://www.fda.gov/drugs/resources-information-approved-drugs/fda-grants-accelerated-approval-zongertinib-non-squamous-nsclc-her2-tkd-activating-mutations?utm_source=chatgpt.com.

[36] FDA, Food and Drug Administration (2025). Oncology (Cancer)/Hematologic Malignancies Approval Notifications. https://www.fda.gov/drugs/resources-information-approved-drugs/oncology-cancerhematologic-malignancies-approval-notifications.

[37] FDA, Food and Drug Administration (2025). Oncology Center of Excellence Guidance Documents. https://www.fda.gov/about-fda/oncology-center-excellence/oncology-center-excellence-guidance-documents.

[38] Feng M, Yang Y, Liao W, Li Q (2022). Cost-effectiveness analysis of tyrosine kinase inhibitors in gastrointestinal stromal tumor: A systematic review. Frontiers in Public Health.

[39] Fu J, Yang Y, Zhu L, Chen Y, Liu B (2022). Unraveling the Roles of Protein Kinases in Autophagy: An Update on Small-Molecule Compounds for Targeted Therapy. Journal of Medicinal Chemistry.

[40] Gelderblom H, Bhadri V, Stacchiotti S, Bauer S, Wagner AJ, van de Sande M (2024). Vimseltinib versus placebo for tenosynovial giant cell tumour (MOTION): a multicentre, randomised, double-blind, placebo-controlled, phase 3 trial. The Lancet.

[41] Gelderblom H, Razak AA, Taylor MH, Bauer TM, Wilky B, Martin-Broto J (2024). CSF1R Inhibition in Patients with Advanced Solid Tumors or Tenosynovial Giant Cell Tumor: A Phase I Study of Vimseltinib. Clin Cancer Res.

[42] Gerber DE, Camidge DR, Morgensztern D, Cetnar J, Kelly RJ, Ramalingam SS (2020). Phase 2 study of the focal adhesion kinase inhibitor defactinib (VS-6063) in previously treated advanced KRAS mutant non-small cell lung cancer. Lung Cancer.

[43] Goranova-Marinova V, Grekova-Kafalova D, Andreevska K, Georgieva V, Gvozdeva Y, Kassarova M (2024). Analysis of the pharmacoeconomic effectiveness of the tyrosine kinase inhibitors therapy in patients with chronic myeloid leukemia in a Single Hematology Center in Plovdiv, Bulgaria. Pharmacia.

[44] Grisham R, Monk B, Nieuwenhuysen E, Moore K, Fabbro M, O'Malley D (2024). A phase III, randomized trial evaluating avutometinib plus defactinib compared with investigator's choice of therapy among patients with recurrent low-grade serous ovarian cancer: GOG-3097/ENGOT-OV81/NCRI/RAMP 301. Gynecologic Oncology.

[45] Grisham R, Monk BJ, Van Nieuwenhuysen E, Moore KN, Fabbro M, O’Malley DM (2025). GOG-3097/ENGOT-ov81/GTG-UK/RAMP 301: a phase 3, randomized trial evaluating avutometinib plus defactinib compared with investigator’s choice of treatment in patients with recurrent low grade serous ovarian cancer. International Journal of Gynecological Cancer.

[46] Gross S, Rahal R, Stransky N, Lengauer C, Hoeflich KP (2015). Targeting cancer with kinase inhibitors. J Clin Invest.

[47] Hao C, Li X, Wang Z, Liu L, He F, Pan Z (2023). Optically activated MEK1/2 inhibitors (Opti-MEKi) as potential antimelanoma agents. European Journal of Medicinal Chemistry.

[48] Heymach JV, Opdam F, Barve M, Tu HY, Wu YL, Berz D (2025). HER2-Selective Tyrosine Kinase Inhibitor, Zongertinib (BI 1810631), in Patients With Advanced/Metastatic Solid Tumors With HER2 Alterations: A Phase Ia Dose-Escalation Study. J Clin Oncol.

[49] Heymach JV, Ruiter G, Ahn M-J, Girard N, Smit E, Planchard D (2025). Abstract CT050: Zongertinib in patients with pretreated HER2-mutant advanced NSCLC: Beamion LUNG-1. Cancer Research.

[50] Heymach JV, Ruiter G, Ahn M-J, Girard N, Smit EF, Planchard D (2025). Zongertinib in Previously Treated HER2-Mutant Non–Small-Cell Lung Cancer. New England Journal of Medicine.

[51] Hill A, Gotham D, Fortunak J, Meldrum J, Erbacher I, Martin M (2016). Target prices for mass production of tyrosine kinase inhibitors for global cancer treatment. BMJ open.

[52] Hoy SM (2025). Mirdametinib: First Approval. Drugs.

[53] Hu C, Dignam JJ (2019). Biomarker-driven oncology clinical trials: key design elements, types, features, and practical considerations. JCO Precision Oncology.

[54] Huang Q, Li Y, Huang Y, Wu J, Bao W, Xue C (2025). Advances in molecular pathology and therapy of non-small cell lung cancer. Signal Transduction and Targeted Therapy.

[55] Hurvitz SA, Kakkar R (2012). Role of lapatinib alone or in combination in the treatment of HER2-positive breast cancer. Breast Cancer (Dove Med Press).

[56] Ismail A, Desai A, Boumber Y (2025). HER2 alterations in non-small cell lung cancer (NSCLC): from biology and testing to advances in treatment modalities. Frontiers in Oncology.

[57] Jemal A, Bray F, Center MM, Ferlay J, Ward E, Forman D (2011). Global cancer statistics. CA: a cancer journal for clinicians.

[58] Jeon H, Wang S, Song J, Gill H, Cheng H (2025). Update 2025: Management of Non‑Small-Cell Lung Cancer. Lung.

[59] Jovanoski N, Vaselenak S, Hogan A, Turki J, Chu Q (2025). Cost-Effectiveness of Adjuvant Alectinib Versus Chemotherapy for Patients with Resectable, ALK-positive Non-small Cell Lung Cancer in Canada. Pharmacoeconomics.

[60] Kannaiyan R, Mahadevan D (2018). A comprehensive review of protein kinase inhibitors for cancer therapy. Expert Rev Anticancer Ther.

[61] Khan SU, Fatima K, Aisha S, Malik F (2024). Unveiling the mechanisms and challenges of cancer drug resistance. Cell Commun Signal.

[62] Killock D (2024). Vimseltinib improves outcomes in tenosynovial giant cell tumour. Nature Reviews Clinical Oncology.

[63] Kisielewska K, Rutkowski P (2025). An evaluation of vimseltinib for treatment of tenosynovial giant cell tumors. Expert Review of Anticancer Therapy.

[64] Kocarnik JM, Compton K, Dean FE, Fu W, Gaw BL, Harvey JD (2022). Cancer Incidence, Mortality, Years of Life Lost, Years Lived With Disability, and Disability-Adjusted Life Years for 29 Cancer Groups From 2010 to 2019: A Systematic Analysis for the Global Burden of Disease Study 2019. JAMA Oncol.

[65] Kossakowski K, Cherniienko A, Zaprutko L, Pawełczyk A (2025). FDA-approved kinase inhibitors in PROTAC design, development and synthesis. J Enzyme Inhib Med Chem.

[66] Ku BM, Sun JM, Lee SH, Ahn JS, Park K, Ahn MJ (2017). An update on biomarkers for kinase inhibitor response in non-small-cell lung cancer. Expert Rev Mol Diagn.

[67] Kudek MR, Adashek JJ, Kurzrock R (2025). Ag(e)nostic precision oncology therapy approvals across the years. Trends Cancer.

[68] Kumar R, Goel H, Solanki R, Rawat L, Tabasum S, Tanwar P (2024). Recent developments in receptor tyrosine kinase inhibitors: A promising mainstay in targeted cancer therapy. Medicine in Drug Discovery.

[69] Kwilas AR, Donahue RN, Tsang KY, Hodge JW (2015). Immune consequences of tyrosine kinase inhibitors that synergize with cancer immunotherapy. Cancer Cell Microenviron.

[70] Lai-Kwon J, Thorner E, Rutherford C, Crossnohere N, Brundage M (2024). Integrating Patient-Reported Outcomes Into the Care of People With Advanced Cancer—A Practical Guide. American Society of Clinical Oncology Educational Book.

[71] Latham BD, Geffert RM, Jackson KD (2024). Kinase Inhibitors FDA Approved 2018-2023: Drug Targets, Metabolic Pathways, and Drug-Induced Toxicities. Drug Metab Dispos.

[72] Li BT, Smit EF, Goto Y, Nakagawa K, Udagawa H, Mazières J (2022). Trastuzumab deruxtecan in HER2-mutant non–small-cell lung cancer. New England Journal of Medicine.

[73] Lim K-H, Safyan RA, Perez K, Spencer KR, O'Reilly EM, Ko AH (2025). Avutometinib/defactinib and gemcitabine/nab-paclitaxel combination in first-line metastatic pancreatic ductal adenocarcinoma: Updated safety and efficacy of a phase 1b/2 study (RAMP 205). J Clin Oncol.

[74] Liu H, Qin J, Qian X (2024). Targeting EGFR Exon 20 Insertion Mutations in Non-small-Cell Lung Cancer: Changes in Treatment Strategies are Coming. Cancer Control.

[75] Lokaj R (2023). Avutometinib/Defactinib Shows Efficacy in Treating Low-grade Serous Ovarian Cancer. Cancer Network.

[76] Lovly CM, Shaw AT (2014). Molecular pathways: resistance to kinase inhibitors and implications for therapeutic strategies. Clin Cancer Res.

[77] Lui A, Vanleuven J, Perekopskiy D, Liu D, Xu D, Alzayat O (2022). FDA-Approved Kinase Inhibitors in Preclinical and Clinical Trials for Neurological Disorders. Pharmaceuticals (Basel).

[78] Malandrini F, Meregaglia M, Di Maio M, Pinto C, De Lorenzo F, Ciani O (2024). The development of an archive of patient-reported outcome measures (PROMs) in oncology: The Italian PRO4All project. European Journal of Cancer.

[79] Mansoori B, Mohammadi A, Davudian S, Shirjang S, Baradaran B (2017). The Different Mechanisms of Cancer Drug Resistance: A Brief Review. Adv Pharm Bull.

[80] McNamara B, Demirkiran C, Hartwich TMP, Bellone S, Manavella D, Mutlu L (2024). Preclinical efficacy of RAF/MEK clamp avutometinib in combination with FAK inhibition in low grade serous ovarian cancer. Gynecol Oncol.

[81] Meng L, Wu H, Wu J, Ding Pa, He J, Sang M (2024). Mechanisms of immune checkpoint inhibitors: insights into the regulation of circular RNAS involved in cancer hallmarks. Cell Death & Disease.

[82] Miller GD, Bruno BJ, Lim CS (2014). Resistant mutations in CML and Ph(+)ALL - role of ponatinib. Biologics.

[83] Mina SA, Shanshal M, Leventakos K, Parikh K (2025). Emerging targeted therapies in non-small-cell lung cancer (NSCLC). Cancers.

[84] Mingione VR, Paung Y, Outhwaite IR, Seeliger MA (2023). Allosteric regulation and inhibition of protein kinases. Biochem Soc Trans.

[85] Mitsudomi T (2025). Sunvozertinib: shining light on lung cancer's exon 20 fight. Transl Lung Cancer Res.

[86] Mukhopadhyay A, Dasgupta S, Mukhopadhyay S, Bose CK, Sarkar S, Gharami F (2012). Imatinib mesylate therapy in patients of chronic myeloid leukemia with Philadelphia chromosome positive: an experience from eastern India. Indian J Hematol Blood Transfus.

[87] Nguyen AN, Hollenbach PW, Richard N, Luna-Moran A, Brady H, Heise C (2010). Azacitidine and decitabine have different mechanisms of action in non-small cell lung cancer cell lines. Lung Cancer (Auckl).

[88] OECD (2024). Tackling the Impact of Cancer on Health, the Economy and Society, OECD Health Policy Studies,.

[89] Opdam F, Heymach J, Berz D, Barve M, Tu H-Y, Wu Y-L (2024). MA12. 10 Zongertinib (BI 1810631) for HER2-positive solid tumors with brain metastases: Subanalysis of the Beamion LUNG-1 trial. Journal of Thoracic Oncology.

[90] Owen DH, Jaiyesimi IA, Leighl NB, Ismaila N, Florez N, Puri S (2024). Therapy for Stage IV Non–Small Cell Lung Cancer With and Without Driver Alterations: ASCO Living Guideline Clinical Insights. JCO Oncology Practice.

[91] Oztosun G, Armstrong A, Hirbe AC (2025). Mirdametinib, an FDA-Approved MEK1/2 inhibitor for adult and pediatric NF1-associated plexiform neurofibromas. Expert Opin Investig Drugs.

[92] Pan C, Olsen JV, Daub H, Mann M (2009). Global effects of kinase inhibitors on signaling networks revealed by quantitative phosphoproteomics. Mol Cell Proteomics.

[93] PDQ Cancer Information Summaries Non-small cell lung cancer treatment (PDQ®) (2025). PDQ cancer information summaries. https://www.ncbi.nlm.nih.gov/books/NBK65917/.

[94] Pe M, Voltz-Girolt C, Bell J, Bhatnagar V, Bogaerts J, Booth C (2025). Using patient-reported outcomes and health-related quality of life data in regulatory decisions on cancer treatment: highlights from an EMA-EORTC workshop. The Lancet Oncology.

[95] Perry MB, Taylor S, Khatoon B, Vercell A, Faivre-Finn C, Velikova G (2024). Examining the Effectiveness of Electronic Patient-Reported Outcomes in People With Cancer: Systematic Review and Meta-Analysis. J Med Internet Res.

[96] Pickard L, Mitsopolous K, Roumeliotis T, Coma S, Hover L, De Haven Brandon A (2025). Correlative preclinical studies to elucidate mechanisms of synergy of the combination of the RAF/MEK clamp avutometinib and the FAK inhibitor defactinib in low grade serous ovarian cancer. Cancer Research.

[97] Puzanov I, Amaravadi RK, McArthur GA, Flaherty KT, Chapman PB, Sosman JA (2015). Long-term outcome in BRAF(V600E) melanoma patients treated with vemurafenib: Patterns of disease progression and clinical management of limited progression. Eur J Cancer.

[98] Qadri M, Ambreen U, Javaid H, Fatima F, Sabir B, Hussain A (2025). FDA approval of vimseltinib (romvimza): a transformative advance for patients with unresectable tenosynovial giant cell tumor. Annals of Medicine and Surgery.

[99] Raggi D, Crupi E, Pederzoli F, Martini A, Briganti A, Alhalabi O (2025). HER2 and urothelial carcinoma: current understanding and future directions. Nat Rev Urol.

[100] Roskoski R (2024). Cost in the United States of FDA-approved small molecule protein kinase inhibitors used in the treatment of neoplastic and non-neoplastic diseases. Pharmacological Research.

[101] Roskoski R (2025). Properties of FDA-approved small molecule protein kinase inhibitors: A 2025 update. Pharmacological Research.

[102] Sacha T (2014). Imatinib in chronic myeloid leukemia: an overview. Mediterr J Hematol Infect Dis.

[103] Sanford D, Kantarjian H, Skinner J, Jabbour E, Cortes J (2015). Phase II trial of ponatinib in patients with chronic myeloid leukemia resistant to one previous tyrosine kinase inhibitor. Haematologica.

[104] Santarpia M, Liguori A, Karachaliou N, Gonzalez-Cao M, Daffinà MG, D’Aveni A (2017). Osimertinib in the treatment of non-small-cell lung cancer: design, development and place in therapy. Lung Cancer: Targets and Therapy.

[105] Sayegh N, Yirerong J, Agarwal N, Addison D, Fradley M, Cortes J (2023). Cardiovascular Toxicities Associated with Tyrosine Kinase Inhibitors. Curr Cardiol Rep.

[106] Seedor RS, Terai M, Majeed A, Tanaka R, Aplin AE, Orloff M (2024). Abstract CT260: A phase II trial of defactinib combined with avutometinib in patients with metastatic uveal melanoma. Cancer Research.

[107] Shalata W, Maimon Rabinovich N, Agbarya A, Yakobson A, Dudnik Y, Abu Jama A (2024). Efficacy of Pembrolizumab vs. Nivolumab Plus Ipilimumab in Metastatic NSCLC in Relation to PD-L1 and TMB Status. Cancers (Basel).

[108] Shin J, Zhang A, Meyer A, Ogle A, Shatara M, Cluster A (2024). LGG-55. Differential toxicity profile of individual MEK inhibitors: a multi-institution retrospective review. Neuro-Oncology.

[109] Shulman LN, Berry DA, Cirrincione CT, Becker HP, Perez EA, O'Regan R (2014). Comparison of doxorubicin and cyclophosphamide versus single-agent paclitaxel as adjuvant therapy for breast cancer in women with 0 to 3 positive axillary nodes: CALGB 40101 (Alliance). J Clin Oncol.

[110] Shyam Sunder S, Sharma UC, Pokharel S (2023). Adverse effects of tyrosine kinase inhibitors in cancer therapy: pathophysiology, mechanisms and clinical management. Signal Transduction and Targeted Therapy.

[111] Singh DD, Lee H-J, Yadav DK (2022). Clinical updates on tyrosine kinase inhibitors in HER2-positive breast cancer. Frontiers in pharmacology.

[112] Smith BD, Kaufman MD, Wise SC, Ahn YM, Caldwell TM, Leary CB (2021). Vimseltinib: A Precision CSF1R Therapy for Tenosynovial Giant Cell Tumors and Diseases Promoted by Macrophages. Mol Cancer Ther.

[113] Son J, Jang J, Beyett TS, Eum Y, Haikala HM, Verano A (2022). A Novel HER2-Selective Kinase Inhibitor Is Effective in HER2 Mutant and Amplified Non-Small Cell Lung Cancer. Cancer Res.

[114] SpringWorks Therapeutics (2023). SpringWorks Therapeutics Announces Positive Topline Results from the Phase 2b ReNeu Trial of Mirdametinib in NF1-PN. https://www.ctf.org/news/springworks-announces-positive-topline-results-from-phase-2b-reneu-trial-of-mirdametinib/.

[115] SpringWorks Therapeutics (2025). SpringWorks Therapeutics Receives Positive CHMP Opinion for Mirdametinib for the Treatment of Adult and Pediatric Patients with NF1-PN. https://springworkstx.gcs-web.com/news-releases/news-release-details/springworks-therapeutics-receives-positive-chmp-opinion.

[116] Stover EH, Lee EK, Shapiro GI, Brugge JS, Matulonis UA, Liu JF (2025). The RAS-MEK-ERK pathway in low-grade serous ovarian cancer. Gynecologic Oncology.

[117] Sun K, Wang X, Zhang H, Lin G, Jiang R (2024). Management and mechanisms of diarrhea induced by tyrosine kinase inhibitors in human epidermal growth factor receptor-2-positive breast cancer. Cancer Control.

[118] Talon B, Calip GS, Lee TA, Sharp LK, Patel P, Touchette DR (2021). Trend in Tyrosine Kinase Inhibitor Utilization, Price, and Out-of-Pocket Costs in Patients With Chronic Myelogenous Leukemia. JCO Oncology Practice.

[119] Tan BK, Bee PC, Chua SS, Chen L-C (2021). Monitoring and improving adherence to tyrosine kinase inhibitors in patients with chronic myeloid leukemia: a systematic review. Patient preference and adherence.

[120] Tap WD, Bhadri V, Stacchiotti S, Bauer S, Wagner AJ, Sande Mvd (2024). Efficacy, safety, and patient-reported outcomes of vimseltinib in patients with tenosynovial giant cell tumor: Results from the phase 3 MOTION trial. Journal of Clinical Oncology.

[121] Tap WD, Sharma MG, Vallee M, Smith BD, Sherman ML, Ruiz-Soto R (2024). The MOTION study: a randomized, phase III study of vimseltinib for the treatment of tenosynovial giant cell tumor. Future Oncol.

[122] Tóth K, Gaál Z (2025). Impact of Tyrosine Kinase Inhibitors on the Expression Pattern of Epigenetic Regulators. Cancers (Basel).

[123] Tucker N (2023). Sunvozertinib Yields Tolerability, Efficacy in EGFR+ NSCLC. Cancer Network.

[124] Vaez-Gharamaleki Y, Hosseini MS (2024). Improved access to the innovative anticancer therapies in resource-limited countries: call for global action. Int J Surg.

[125] Verdin P (2024). FDA new drug approvals in Q2 2024. Nature Reviews Drug Discovery.

[126] Verschueren MV, Hiensch DTA, Plomp PMJ, Kastelijn LA, van de Garde EMW, Peters BJM (2025). Comparative Effectiveness of Nivolumab and Ipilimumab Plus Chemotherapy Versus Pembrolizumab Plus Chemotherapy in PD-L1 Negative Metastatic Non-Small Cell Lung Cancer Patients. Clinical Lung Cancer.

[127] Wagner AJ, D’Amato G, Ganjoo K (2024). Safety, efficacy, and patient-reported outcomes with vimseltinib in patients with tenosynovial giant cell tumor who received prior anti–colony-stimulating factor 1 therapy: ongoing phase 2 update. Knee.

[128] Wang B, Wu H, Hu C, Wang H, Liu J, Wang W (2021). An overview of kinase downregulators and recent advances in discovery approaches. Signal Transduction and Targeted Therapy.

[129] Wang M, Fan Y, Sun M, Wang Y, Zhao Y, Jin B (2024). Sunvozertinib for patients in China with platinum-pretreated locally advanced or metastatic non-small-cell lung cancer and EGFR exon 20 insertion mutation (WU-KONG6): single-arm, open-label, multicentre, phase 2 trial. Lancet Respir Med.

[130] Wang M, Xu Y, Huang W-T, Su W-C, Gao B, Lee CK (2025). Sunvozertinib monotherapy in EGFR tyrosine kinase inhibitor-resistant non-small cell lung cancer with EGFR mutations. Lung Cancer.

[131] Wang M, Yang JC-H, Mitchell PL, Fang J, Camidge DR, Nian W (2022). Sunvozertinib, a selective EGFR inhibitor for previously treated non–small cell lung cancer with EGFR exon 20 insertion mutations. Cancer Discovery.

[132] Weiss BD, Wolters PL, Plotkin SR, Widemann BC, Tonsgard JH, Blakeley J (2021). NF106: a neurofibromatosis clinical trials consortium phase II trial of the MEK inhibitor mirdametinib (PD-0325901) in adolescents and adults with NF1-related plexiform neurofibromas. Journal of Clinical Oncology.

[133] WHO, World Health Organization (2002). National cancer control programmes: policies and managerial guidelines. https://www.who.int/publications/i/item/national-cancer-control-programmes.

[134] Wilding B, Woelflingseder L, Baum A, Chylinski K, Vainorius G, Gibson N (2025). Zongertinib (BI 1810631), an Irreversible HER2 TKI, Spares EGFR Signaling and Improves Therapeutic Response in Preclinical Models and Patients with HER2-Driven Cancers. Cancer Discov.

[135] Woudberg R, Sinanovic E (2024). Cost-effectiveness of tyrosine kinase inhibitor treatment strategies for chronic myeloid leukemia in South Africa. Front Pharmacol.

[136] Wu Y-L, Opdam F, Yamamoto N, Yoshida T, Heymach J (2024). P3. 12D. 06 Beamion LUNG-1 And LUNG-2: The Zongertinib Clinical Program in Patients with Non-Small Cell Lung Cancer and HER2 Mutations. Journal of Thoracic Oncology.

[137] Wynn ML, Ventura AC, Sepulchre JA, García HJ, Merajver SD (2011). Kinase inhibitors can produce off-target effects and activate linked pathways by retroactivity. BMC Syst Biol.

[138] Xie K, Wang J, Jiang J, Deng Z, Hu Q, Wang D (2025). Efficacy and safety outcomes of emerging EGFR‑TKIs for patients with non‑small cell lung cancer with EGFR exon 20 insertion mutations: A systematic review and meta‑analysis. Oncology Letters.

[139] Xu Y, Chen M, Gao X, Liu X, Zhao J, Zhong W (2025). Efficacy and safety of sunvozertinib monotherapy as first-line treatment in NSCLC patients with EGFR exon 20 insertion mutations: A phase 2, single-center trial. Cancer Letters.

[140] Yang JC-H, Doucet L, Wang M, Fan Y, Sun M, Greillier L (2024). A multinational pivotal study of sunvozertinib in platinum pretreated non-small cell lung cancer with EGFR exon 20 insertion mutations: Primary analysis of WU-KONG1 study. Journal of Clinical Oncology.

[141] Yegnasubramanian S, Maitra A (2013). Aiming for the outliers: cancer precision medicine through targeting kinases with extreme expression. Cancer Discov.

[142] Zafar A, Khatoon S, Khan MJ, Abu J, Naeem A (2025). Advancements and limitations in traditional anti-cancer therapies: a comprehensive review of surgery, chemotherapy, radiation therapy, and hormonal therapy. Discover oncology.

[143] Zeng J, Ma W, Young RB, Li T (2021). Targeting HER2 genomic alterations in non-small cell lung cancer. Journal of the National Cancer Center.

[144] Zhou F, Guo H, Xia Y, Le X, Tan DSW, Ramalingam SS (2025). The changing treatment landscape of EGFR-mutant non-small-cell lung cancer. Nature Reviews Clinical Oncology.

[145] Zohourian N, Brown JA (2024). Current trends in clinical trials and the development of small molecule epigenetic inhibitors as cancer therapeutics. Epigenomics.

